# Hospital Intervention to Reduce Overweight with Educational Reinforcement after Discharge: A Multicenter Randomized Clinical Trial

**DOI:** 10.3390/nu14122499

**Published:** 2022-06-16

**Authors:** Carmen Herrera-Espiñeira, María del Carmen Martínez-Cirre, Manuel López-Morales, Antonia Lozano-Sánchez, Antonia Rodríguez-Ruíz, Laura Esther Salmerón-López, María Isabel Gómez-Crespo, Manuela Expósito-Ruíz

**Affiliations:** 1 Faculty of Health Sciences, University of Granada, 18012 Granada, Spain; malomorales@ugr.es; 2 Instituto de Investigación Biosanitaria (ibs.GRANADA), 18012 Granada, Spain; 3 National Network of Research in Health Departments and Chronic Diseases (REDISSEC), 18012 Granada, Spain; 4 Clinical Documentation Unit, Virgen de las Nieves University Hospital, 18014 Granada, Spain; carmen.martinez.cirre.sspa@juntadeandalucia.es; 5 Granada-Metropolitan Health District, 18012 Granada, Spain; 6 Department of Internal Medicine, Baza Hospital, 18800 Baza, Spain; antonia.lozano.sanchez.sspa@juntadeandalucia.es; 7 Department of Internal Medicine, Motril Hospital, 18600 Motril, Spain; antoniarruiz@hotmail.com; 8 Department of Internal Medicine, Virgen de las Nieves University Hospital, 18014 Granada, Spain; lauraesl84@gmail.com; 9 Ceuta University Hospital, 51003 Ceuta, Spain; maycre@hotmail.com; 10 Unit of Biostatistics, Department of Statistics, School of Medicine, University of Granada, 18012 Granada, Spain; mexpositoruiz@ugr.es

**Keywords:** overweight, exercise, food habits, patients, internal medicine, clinical trial

## Abstract

Introduction: Obesity and overweight affect more than one-third of the world’s population and pose a major public health problem. Objective: To evaluate the impact of an educational intervention on dietary habits and physical exercise in patients with overweight admitted to departments of internal medicine, comprising a pre-discharge educational session with follow-up and reinforcement by telephone at 3, 6, and, 12 months post-discharge. Outcome variables were weight, systolic (SBP) and diastolic (DBP) blood pressures, health-related quality of life (HRQOL), hospital readmissions, emergency department visits, and death. Method: A randomized experimental study with a control group was performed in hospitalized non-diabetic adults aged ≥18 years with body mass index (BMI) ≥25 Kg/m^2^. Results and conclusions: The final sample included 273 patients. At three months post-discharge, the intervention group had lower SBP and DPB and improved dietary habits (assessed using the Pardo Questionnaire) and VAS-assessed HRQOL in comparison to the control group but a worse EQ-5Q-5L-assessed HRQOL. There were no between-group differences in hospital readmissions, emergency department visits, or mortality at any time point. Both groups evidenced a progressive improvement over the three follow-up periods in weight, SBP, and dietary habits but a worsening of EQ-5D-5L-value-assessed HRQOL. Discussion: The intervention group showed greater improvements over the short term, but between-group differences disappeared at 6 and 12 months. Weight loss and improvements in key outcomes were observed in both groups over the follow-up period. Further research is warranted to determine whether a minimum intervention with an educational leaflet, follow-up phone calls, and questionnaires on overweight-related healthy habits, as in the present control group, may be an equally effective strategy without specific individual educational input.

## 1. Introduction

Overweight and obesity have a major impact on health [1] and health-related quality of life (HRQOL) worldwide, with important socio-economic consequences [2,3]. It was estimated by the WHO that the worldwide prevalence of obesity tripled between 1975 and 2016, when 39% of adults were with overweight and 13% with obesity [1]. In 2005, Spain implemented a Strategy for Nutrition, Physical Activity, and the Prevention of Obesity (NAOS) in line with WHO recommendations [4]; however, the alarming increase of obesity in Spain has not diminished, and its prevalence was 2.4-fold higher in 2017 (17.4%) than in 1987 (7.4%) [5]. The WHO describes the main cause of obesity and overweight as an energy imbalance between calories consumed and expended [6]. The rise in overweight/obesity might therefore be explained by worldwide increases in the intake of high-calorie food and sedentariness, although complex interactions among biological, behavioral, social, and environmental factors are involved [7].

Primary care interventions to combat overweight and obesity have varied in terms of approach and effectiveness. Literature reviews [8,9] have concluded that programs addressing both physical activity and health behavior are more effective over the long term than those that focus on behavior. Studies have always considered weight change and sometimes blood pressure (BP) and anthropometric or metabolic variables; however, they have not investigated the utilization of healthcare services or mortality rates.

Other health education programs started during hospitalization have proven effective, including one for trauma surgery patients that reduced visits to the emergency department [10]; a program (PRIC study) for patients with heart failure, which improved their adherence to treatment, HRQOL, and readmission and mortality rates [11]; and an intervention on smoking cessation during the hospital stay [12]. One study in the primary care setting reported a significant reduction in body mass index (BMI) at six months after a single 15 min session of advice on diet and physical activity in patients with overweight, with no subsequent reinforcement [13]. 

With this background, we hypothesized that a simple low-cost educational intervention carried out by nurses, with telephone follow-up calls by a psychologist over a one-year period, would be effective in patients with overweight whose experience of an acute process might generate a favorable attitude towards changing their overweight-related habits. Moreover, initiation of the program during the hospitalization of patients could be expected to facilitate their enrolment. The objective of this study was to evaluate the impact of an educational intervention for hospitalized patients with overweight/obesity to promote a healthy diet and physical activity on their post-discharge weight, BP, overweight-related healthy habits, HRQOL, and other variables less frequently considered in studies, including readmissions, visits to the emergency department, and deaths related to their admission diagnosis. Patients were recruited from departments of internal medicine (IM), whose patients frequently have comorbidities related to overweight and obesity.

## 2. Materials and Methods

This randomized experimental study with control group followed CONSORT guidelines for the assessment of non-pharmacological treatments (Supplementary Materials Table S1). It included adults with overweight admitted to the IM departments of four Spanish hospitals. Eligible patients were randomly assigned to the intervention or control group at admission. The study period was from 26 February 2018 to 25 February 2020, with 24 February 2021 being the end date of the follow-up for the most recently enrolled patients. 

### 2.1. Inclusion and Exclusion Criteria

Inclusion criteria were age ≥18 years with BMI ≥ 25 kg/m^2^ at hospital admission (Virgen de las Nieves University Hospital, Granada; Baza Regional Hospital; Motril Regional Hospital; and University Hospital of Ceuta). Exclusion criteria were: diagnosis of diabetes before or during hospitalization; cognitive/physical status hampering completion of questionnaires or fulfillment of physical exercise or dietary recommendations even with the assistance of a caregiver, according to the criteria of the principal collaborating nurse; pre-admission weight-loss diet controlled by nutritionist/endocrinologist; the receipt of major surgery during the hospital stay; and the refusal of consent to participation. Diabetics were specifically excluded to avoid interference with or influence from the multidisciplinary plan for diabetes of the regional health ministry [14].

### 2.2. Sample

There appears to be a consensus that a weight loss of ≥5% yields important benefits for cardiovascular health [15]. The sample size was calculated to detect a between-group difference of ≥7% with a statistical power of 80% and significance level of 5%. It was estimated that each group should contain at least 248 patients. The actual sample size achieved (*n* = 273) offered a power of 98%, 82%, and 88%, respectively, to detect this difference in systolic blood pressure, (SBP), diastolic blood pressure (DBP), and healthy eating (HD) but a power below 80% for the other secondary outcome variables. Patients meeting the eligibility criteria were consecutively enrolled in the study. The randomized allocation to the intervention or control group was supervised in each center by a nurse unconnected to the research who reported results to the collaborating nurse at the center. The allocation was carried out using a computer-generated sequence of random numbers. Participants, caregivers, and evaluators were blinded to the group membership of the patients, with codes being used to label their data.

### 2.3. Evaluation Protocol and Study Variables 

Two specifically trained registered nurses were involved in the project at each center: a principal collaborating nurse and an assistant collaborating nurse. At hospital admission, the former recorded the sex and age of eligible patients and measured their weight (P_o_), height (barefoot and in pajamas), and systolic (SBP) and diastolic (DBP) blood pressures, always using the appropriate calibrated instruments available in their IM department. This nurse also gathered data from patients on their schooling (primary/none, secondary, or higher education), work situation (unemployed, actively employed, student, retired, or permanent disability), the population of their municipality of residence (≤20,000/>20,000 inhabitants), cohabitation status (living alone or accompanied), smoking habit (yes/no), pre-admission religion-based diet (yes/no), pre-admission medication for anxiety or depression (anxiolytics and/or antidepressants) (yes/no) due to the bidirectional relationship between depression and obesity [16], participation in any previous weight-loss program (yes/no), and the main diagnosis at admission. All participants also completed the Spanish adaptation [17] of the Charlson Combined Comorbidity Index (CCI) questionnaire [18], the Pardo questionnaire on pre-admission diet and physical exercise [19], and the International Physical Activity Questionnaire (IPAQ) on preadmission activity [20]. 

On the day before the hospital discharge of patients, the principal collaborating nurse opened the envelope containing the sequence of randomized numbers that assigned patients to the intervention or control group and informed the assistant collaborating nurse of the group membership of patients. Patients in the intervention group then underwent a one-to-one bedside educational session (10–15 min) with the assistant collaborating nurse, as detailed below, while those in the control group were given an information leaflet summarizing the main points (Supplementary File S1).

At hospital discharge, the assistant collaborating nurse at each center recorded the days of hospital stay and administered the validated Spanish adaptation [21] of the EQ-5D-5L quality of life questionnaire, including the visual analog scale (VAS) [22]. Participants were reminded that they should have their weight, SBP, and DBP measured within the next two days at their local licensed pharmacy, where this service is offered free on demand. They were also reminded by the nurse that he/she would contact them by telephone on that day for the results and that they would again be contacted, by a psychologist, at 3, 6, and 12 months for the same measurements taken at the pharmacy under similar conditions (timing and clothing), when they would also be asked to complete the questionnaires administered at hospital admission. 

Intervention: In a one-to-one session on the day before their discharge, the patient (with caregiver when necessary) received advice on healthy eating and physical activity and on the potential repercussions of overweight on health. The session lasted 10–15 min, and a tablet was used for audiovisual support [23]. The session was called “*Education of the 4 Cs*”, the initials of the Spanish words for Buying, Cooking, Eating, and Walking (*Comprar*, *Cocinar*, *Comer*, and *Caminar*), which comprised the four main components of the session (Supplementary File S2). Relevant points were reinforced during follow-up phone calls in the intervention group alone (see below).

At 3, 6, and 12 months post-discharge, a single psychologist contacted all participants by phone to record their weight and BP measurements and administer the questionnaires. At these follow-up sessions, participants in the intervention group received reinforcement of the information given in the educational session based on a comparison between the patient’s questionnaire findings and previous results. Participants in the control group received no explicit reinforcement. 

At the end of the study, a medical documentalist gathered all data on readmissions, visits to the emergency department, and deaths related to the admission diagnosis (outcome variables) from the electronic health records of the Andalusian (DIRAYA) and Ceuta Health Systems. 

The primary outcome variable was the weight, and the secondary outcome variables were Pardo Questionnaire dimension results, IPAQ questionnaire-measured physical activity, EQ-5D-5L value, and VAS of the EQ-5D-5L, and readmissions, visits to the emergency department, and deaths related to admission diagnosis.

### 2.4. Measurement Instruments

The CCI [17] estimates the risk of death as a function of comorbidities, considering 19 diseases with scores between 1 and 6 points and adding 1 point for each decade past the age of 40 years.

The Pardo Questionnaire [19] was used to quantify pre-admission life habits related to overweight and obesity. It includes 22 questions grouped in five dimensions/factors: (1) dietary caloric intake (CC dimension), with 8 questions; (2) physical activity (PE dimension), with 3 questions; (3) healthy eating (HD dimension), with 6 questions; (4) alcohol intake (AC dimension), with 2 questions; and (5) eating for psychological well-being (PW dimension), with 2 questions. The five response options—never, rarely, sometimes, often, and always—are available for all items, except for the two items “consume drinks with low alcohol content (beer, wine)” and “consume drinks with high alcohol content (liqueurs, gin, whisky)”, to which the responses are never, once a month, once a week, several times a week, or every day. Each question has five response options (1 to 5), with higher scores indicating better CC, HD, and PE results and worse AC and PW results. Questions with reverse scores were transformed before calculating the dimension results, so that higher scores always indicated superior habits/wellbeing status.

The short version of the IPAQ [20] was administered, designed for ages between 15 and 69 years. It contains four generic items and evaluates activity level as low, moderate, or high (in MET units): low activity = no or mild physical activity; moderate activity = ≥3 days of vigorous physical activity of ≥20 min/day or ≥5 days of moderate physical activity and/or walking for ≥30 min/day or ≥5 days of any combination of activity and walking (total of ≥600 MET-min/week); and high activity = activity of vigorous intensity for ≥3 days (≥1500 MET-min/week), or ≥7 days of any combination of walking and activities of moderate or vigorous intensity (≥3000 MET-min/week).

The EQ-5D-5L Questionnaire (EuroQol) [21] measures self-perceived health on the day of its completion and comprises: a descriptive section with five dimensions (mobility, personal care, daily activities, pain/discomfort, and anxiety/depression), each with five response options (higher score = worse status); and a VAS for self-perceived health status (0 = worst to 100 = best imaginable). Questionnaire dimension values are used to obtain the global EQ-5D-5L value (between 0 (worst health status) and 1 (optimal status)), with even the possibility of a negative value (health perceived as worse than death).

Responses to EuroQol and IPAQ questionnaires were considered in the descriptive statistical analysis; however, they were not compared between groups because the statistical power was insufficient for this purpose.

Diagnoses at admission were grouped according to the 10th revision of the International Classification of Diseases (IDC10) [24].

### 2.5. Statistical Analysis

In a descriptive analysis, numerical values were expressed as medians with interquartile range and 25th and 75th percentiles (IQR (P25–P75)) due to their non-normal distribution (Shapiro–Wilk test). Categorical values were expressed as absolute numbers (*n*) and relative (%) frequencies. In bivariate analyses, baseline characteristics were compared between intervention and control groups using Pearson’s chi-square test with continuity correction or, when conditions were not met (not more than 20% of expected frequencies <5), Fisher’s exact test for qualitative variables, using the non-parametric Mann–Whitney test for numerical variables. Changes in weight and the other variables of interest were studied by constructing a general linear model (GLM) with repeated measures, with time as within-subject factor and group as between-subject factor, also analyzing time*group interactions. The Bonferroni test was used for post hoc comparisons to identify the pairs responsible for differences. Means with standard deviations were reported for the GLM. The McNemar test was used to compare IPAQ results (low, moderate, and high activity), EQ-5D-5L questionnaire dimensions, and follow-up events (readmissions, visits to the emergency department, and deaths) among the different time points. The last observation carried forward (LOCF) method was used to treat data for participants lost to the follow-up. IBM SPSS Statistics version 19 was used for data analyses, and *p* < 0.05 was considered significant in all tests.

### 2.6. Ethical Considerations

The study complied with EU regulations (2016/679) and Spanish legislation (3/2018) on personal data protection and digital rights and was conducted in accordance with the 2013 revision of the Declaration of Helsinki (https://www.wma.net/what-we-do/medical-ethics/declaration-of-helsinki/; accessed on 13 July 2017). All subjects gave their informed consent to participate in the study, which was approved by the clinical research ethics committees of Andalusia and the four participating hospitals.

**Figure 1:**
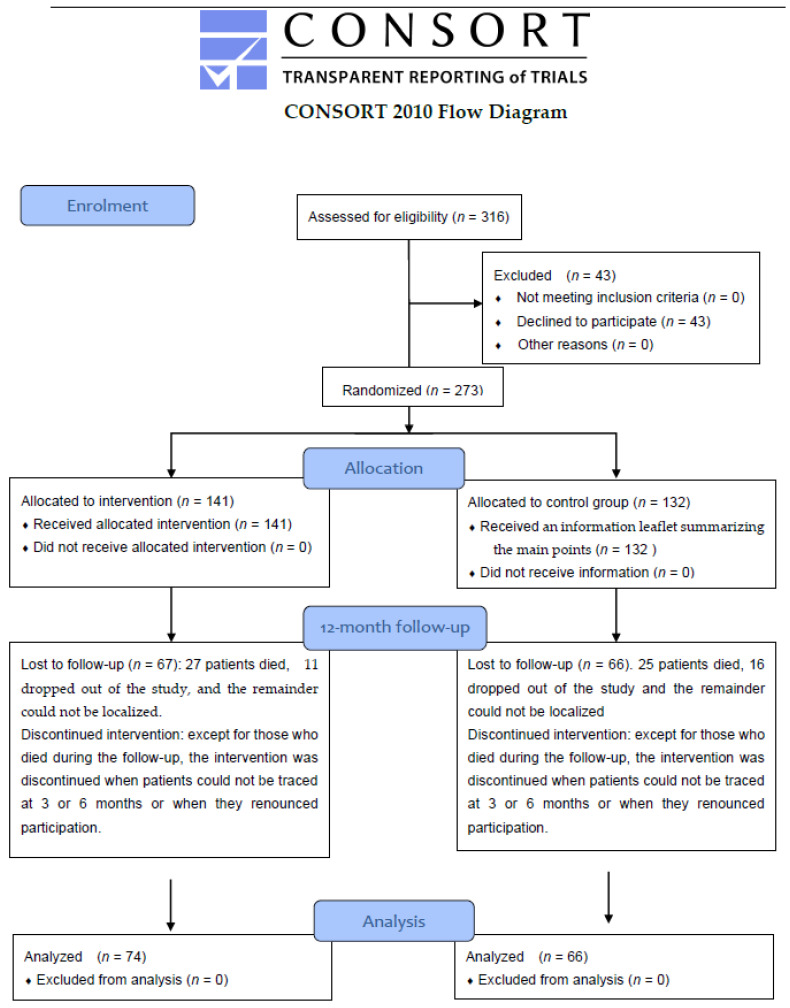
Flow chart of the study.

Below is the flow diagram of the study (Figure 1).

## 3. Results

During the two-year recruitment period, the eligibility criteria were met by 316 patients. Study participation was declined by 43 patients, leaving a final sample of 273 patients, of whom 141 were randomly assigned to the intervention group and 132 to the control group. Out of the 273 patients, 140 completed the 12-month follow-up study—a completion rate of 51.28%; out of the remaining patients, 52 died during the follow-up, 27 dropped out of the study, and the remainder could not be contacted despite repeated attempts.

Table 1 lists the baseline characteristics of the two groups. No significant between-group difference was found in any study variable except for a slightly but significantly higher CCI (median of 2 vs. 3 in controls) and DBP (median of 75 vs. 70) in the intervention group.

As observed in Table 1, the age of patients was similar between the groups (median of 69 yrs in the intervention group and 71.5 yrs in the controls) and both contained a slightly higher percentage of males than females and a higher percentage with primary schooling than with higher educational level or no schooling. More than half of participants in both groups resided in municipalities with ≤20,000 inhabitants, whereas a minority lived alone, had previously undergone a weight-loss program, and reported pre-admission consumption of antidepressants and anxiolytics. The median hospital stay was eight days in both groups.

At hospital discharge, there was no statistically significant difference (*p* = 0.511) in the percentage of patients with BMI ≥30 kg/m^2^ between the intervention (66.7%) and control (62.1%) groups.

Table 2 exhibits the distribution of the main diagnoses (one per patient) responsible for the hospital admissions, showing no significant differences between the intervention and control groups. In both groups, the most frequent diagnoses were related to “other diseases of organs and body systems” (GOO-N99).

No significant between-group differences were found (Pearson’s chi-square test, *p* = 0.409) in the most frequent diagnostic groups (G00–N99 and R00–Z99).

### 3.1. Comparison of Outcome Variables between Baseline (Two Days Post-Discharge) and Three Months

At three months post-discharge, the weight of patients significantly decreased in both groups (*p* < 0.001), with a mean loss of 3.53 kg in the intervention group and 1.77 kg in the control group. However, there was no significant time*group effect (*p* = 0.170). There was a statistically significant time*group effect on SBP (*p* < 0.001) and DBP (*p* = 0.042) values, which decreased in both groups, with a greater reduction in the intervention group (Table 3).

The Pardo questionnaire results showed: a significant improvement in the *CC* dimension over time in both groups (*p* < 0.001), with no significant time*group effect (*p* = 0.212); a significant time*group effect in the PW dimension (*p* = 0.030), with an increase (superior wellbeing) in the intervention group but a decrease in the control group; a significant increase in the HD dimension in both groups (*p* < 0.001) and a significant time*group effect, with a greater increase in the intervention group (*p* = 0.017); and a significant time*group effect on the AC dimension, with an improvement in the intervention group but a worsening in the control group (*p* = 0.021) (Table 3).

In relation to the HRQOL, the EQ-5D-5L score significantly worsened over time (*p* = 0.035) in both groups, whereas the VAS score improved in both groups (*p* = 0.046) (Table 3). Regarding IPAQ results at three months, the intervention group (*n* = 134) showed an increase in the percentage with low activity from 70.1% before admission to 71.6% at three months and in the percentage with moderate activity from 22.4 to 23.9%, whereas the percentage with high activity decreased from 7.5 to 4.5%; these differences were statistically significant (*p* = 0.024). At three months, the percentage of the control group (*n* = 124) with low activity passed from 68.5 to 73.4%, the percentage with moderate activity from 23.9 to 21.8%, and the percentage with high activity from 10.5 to 4.8%; these differences were not statistically significant (*p* = 0.076).

### 3.2. Comparison of Outcome Variables between Baseline and Six Months

As observed in Table 4, no significant between-group differences were observed in the changes in outcome variables between baseline and six months. As at three months, both groups showed significant improvements between baseline and six months in weight loss (moderate loss of 4.2 kg, 2.71 kg in the controls), SBP, DBP, CC, HD, and PE dimensions of the Pardo questionnaire, and VAS score but a worsening of EQ-5D-5L values (Table 4).

According to the IPAQ results (not shown in the table), neither group showed a significant change in low, moderate, or high activity between baseline and six months. In the intervention group, the percentage with low activity passed from 70.1% before admission to 76.1% at six months, the percentage with moderate activity from 22.4 to 14.2%, and the percentage with high activity from 7.5 to 9.7% (*p* = 0.135). In the control group, the percentage with low activity passed from 68.5 to 71%, the percentage with moderate activity from 21 to 24.2%, and the percentage with high activity from 10.5 to 4.8% (*p* = 0.191).

### 3.3. Comparison of Outcome Variables between Baseline and 12 Months

There were no significant between-group differences at 12 months, and both groups showed significant improvement versus the baseline in weight loss (mean decrease of 3.15 kg in the intervention group and 3.73 kg in the controls), SBP, CC, and HD. The difference with observations at six months was that DBP, PE, and VAS results no longer significantly differed from baseline values, while the significantly worse EQ-5D-5L values persisted (Table 5).

In the intervention group, the percentage of patients with low activity passed from 70.1% pre-admission to 76.9% at 12 months, the percentage with moderate activity from 22.4 to 21.6%, and the percentage with high activity from 7.5 to 1.5%, but these differences were only close to statistical significance (*p* = 0.081). In the control group, the percentage with low activity increased (68.5 to 83.9%) and the percentages with moderate (21 to 14.56%) and high (10.5 to 1.6%) activity decreased, as in the intervention group, and these differences were statistically significant (*p* = 0.001).

### 3.4. Readmissions, Visits to Emergency Department, and Deaths during the Follow-up

No significant between-group differences were found in the percentage of readmissions, emergency department visits, or deaths during the follow-up period, as detailed in Table 6.

### 3.5. Analysis Stratified by CCI

Given the differences in baseline CCI values between the intervention and control groups, a stratified analysis of the results at 12 months was conducted, dividing patients between those with low (CCI < 3) and high (CCI ≥ 3) index scores. A CCI score >3 was observed at admission in 39.6% of patients in the intervention group versus 73.3% in the control group (*p* < 0.001). The difference in score between baseline and 12 months was similar between patients with CCI < 3 (*n* = 119) and the global sample. In the patients with CCI < 3, there was no significant time*group effect but a significant time effect on weight loss (mean loss of 4.89 kg vs. 4.4 kg in the control group) (*p* = 0.010), with a weight loss of 5.48% in the intervention group and 4.53% in the control group. An increase (improvement) in CC (*p* < 0.001) and HD (*p* < 0.001) dimensions was observed in both groups, with a statistically significant reduction in physical activity in the control group (IPAQ questionnaire) (*p* = 0.046). There were no significant differences in EQ-5D-5L values or VAS, readmissions, visits to the emergency department, or deaths.

In patients with CCI ≥ 3 (*n* = 151) (Table 7), there was no significant time*group effect. There was no statistically significant between-group difference in weight loss. Both groups showed a significant reduction in SBP and DBP, an improvement in CC and HD, and a worsening in AC. The EQ-5D-5L values significantly worsened in both groups, although there was no change in VAS. There were no significant between-group differences in readmissions, visits to the emergency department, or deaths.

## 4. Discussion

This educational intervention in hospitalized patients with overweight or obesity achieved significant improvements in SBP, DBP, PW, HD, and AC, but these significant differences disappeared at three and six months post-discharge. When only patients with fewer comorbidities (CCI < 3) were considered (*n* = 119), weight loss was significantly greater in the intervention group (5.48%) than in the controls (4.53%), being over the threshold of 5% reported to reduce cardiovascular risk [15].

The same Pardo questionnaire was used in a study by Arrebola et al. with no control group [25], which evaluated the effects of 11 fortnightly health education sessions in primary care centers; they observed an improvement at six months in HD and PE dimensions but a worsening in PW and AC dimensions. The present finding of a reduction in BP at three months versus controls is in line with previous observations [26,27]. The intervention had no effect on the mortality rate, as also observed in the four trials reviewed by LeBlanc et al. [28]. However, the present results in the intervention group are not in agreement with their finding, based on 89 trials of behavioral interventions, of a greater improvement in weight loss at 12 months post-discharge in comparison to controls, as also achieved by eight of the nine interventions reviewed by Taylor et al. in 2013 [29].

In fact, both groups showed significant improvements at 3, 6, and 12 months in weight loss, SBP, CC, and HD, despite the negative effects of the ongoing COVID-19 pandemic on outside physical activity and diet during some of the follow-up. The pandemic, which coincided with the follow-up of around one-third of patients in both groups, was implicated in a worsening of the diet and an increase in the sedentarism of 44% of the Spanish population during its first year [30]. The improvements observed in the control group may indicate a positive effect of the administration (by telephone) of the questionnaires on overweight-related healthy habits at 3, 6, and 12 months. The telephone contact with a psychologist may have encouraged individuals to reflect on their dietary habits and exercise and allowed them to discuss their concerns. In this sense, the study design meant that the control patients were themselves subject to a repeated “intervention”. A psychologist was chosen for this phase of the intervention due to the known relevance of mental state to obesity [16] and because of the need to respond to points raised on the telephone by members of the intervention group on questions in the anxiety/depression and psychological wellbeing dimensions of the questionnaires. However, it should be taken into account that ethical considerations meant that the psychologist could not avoid responding to this type of point when raised by a member of the control group. Leblanc et al. [31] administered the Three-Factor Eating Questionnaire (TFEQ) and also found no significant time*group effect but an improvement in weight reduction and energy intake in both the intervention and control groups. These findings suggest that contacting patients by telephone in this way may improve the feeling of wellbeing in patients and their families [32,33]. Placebo and nocebo effects are related to the positive and negative expectations of patients towards a therapy, in part explaining their response to treatment [34], and these effects have been described in weight loss programs [35] and other studies [36]. In future studies, hopefully with no pandemic restrictions, it may be useful to administer questionnaires to the control group at baseline and 12 months alone to minimize possible confounding effects of their administration at three and six months. It is also important to determine the degree to which these patients are motivated by the severe event causing hospital admission to take measures to lose weight over the year after their discharge. We have been unable to trace published data on this issue.

In both groups, quality of life was worse at the three measurement time points according to EQ-5D-5L values but was improved according to the self-assessed VAS. The authors of [37] reported a weak correlation between VAS and EQ-5D-5L values, concluding that VAS measures a wider underlying condition that is closer to the perspective of patients at the time of assessment in comparison to EQ-5D-5L value, which captures the assessment in a more disaggregated manner. It is also possible that positive expectations encouraged by the provision of information at discharge and by the telephone contacts might in part explain this discrepancy between EQ-5D-5L and VAS values.

Results obtained over the follow-up period did not differ when the groups were stratified by comorbidity burden, except that SBP and DBP were significantly lower, EQ-5D-5L values were significantly worse, and the AC was also significantly worse at 12 months in both groups among the patients with more comorbidities (CCI ≥ 3). In contrast, there was no significant improvement in SBP or DBP or worsening of EQ-5D-5L values in either group among those with fewer comorbidities (CCI < 3), although both groups evidenced significant weight loss, which was above 5% in the intervention group, which is considered to be beneficial for cardiovascular health (15).

### Study Limitations

A major study limitation was the failure to reach the estimated sample size, despite extending the originally planned one-year enrolment period for a further year. One reason was the unexpectedly large proportion of patients who had to be excluded for the presence of diabetes or incapacity to complete the questionnaires due to cognitive impairment. In addition, it is known to be challenging to enroll and retain patients in this type of study, in which only around 53% of patients are reported to complete follow-up periods of ≥12 months [38]. This was especially problematic in the present study because of the coronavirus pandemic, with the consequent negative effects on recruitment and follow-up adherence. Despite these obstacles, only 81 participants (29.67%) dropped out of the follow-up or could not be localized at 12 months, which may be attributable to the strenuous efforts made to contact patients and caregivers and avoid losses to the follow-up. However, studies with larger patient samples and a longer follow-up period are needed to allow conclusions to be drawn.

A different nurse was responsible for data gathering in each center, although all were trained for this purpose by the same researcher and instructed to follow a standard protocol for the measurement of weight/height, BP, and other variables, minimizing possible variability.

## 5. Conclusions

The intervention was effective at three months, reducing both SBP and DBP and improving some aspects of overweight-related dietary habits and health self-assessment with VAS; however, there was a worsening of EQ-5D-5L-assessed HRQOL. Both groups (intervention and control) showed improvements over time (at 3, 6, and 12 months) in weight, SBP, and some aspects of their dietary habits, possibly due to the benefits of the administration of questionnaires on overweight-related healthy habits in both groups during the three follow-up calls or to a placebo effect, although EQ-5D-5L values worsened. Further research is warranted to determine whether a minimum intervention with an educational leaflet, follow-up phone calls, and questionnaires on overweight-related healthy habits, as in the present control group, may be an equally effective strategy without specific individual educational input.

## Figures and Tables

**Table 1:** Comparison of characteristics between intervention and control groups.

Variable	Intervention *n* 141(51.6%)	Control *n* 132(48.4%)	*p*-Value
Age (median (P_25_–P_75_))	69 (53.5–80)	71.5 (58–81.75)	0.140
Sex (men)	75 (53.2%)	73 (55.3%)	0.819
Schooling			
None	37 (26.4%)	43 (33.6%)	0.419
Primary	68 (48.6%)	58 (45.3%)	
Secondary/university	35 (25%)	27 (21.1%)	
Work situation			
Unemployed	15 (10.6%)	11 (8.7%)	0.651
Actively employed/student	28 (19.9%)	21 (16.7%)	
Retired/permanent disability	98 (69.5%)	97 (74.6%)	
Municipality of residence			
≤20,000 inhabitants	78 (54.3%)	72 (55.4%)	1
>20,000 inhabitants	63 (44.7%)	58 (44.6%)	
Cohabitation (lives alone)	21 (15.1%)	13 (10.2%)	0.315
Smoker (yes)	14 (9.9%)	18 (14%)	0.405
Practices religion-based diet (yes)	17 (13.2%)	18 (16.4%)	0.610
Takes anti-depression drugs (yes)	15 (10.9%)	15 (11.8%)	0.962
Takes anxiolytics drugs (yes)	16 (11.5%)	13 (10.2%)	0.892
Concerning problems (yes)	39 (27.9%)	43 (34.4%)	0.309
Previous obesity program	20 (14.4%)	25 (19.8%)	0.309
Days of stay (median (P_25_–P_75_))	8 (6–10)	8 (5–10.25)	0.831
Charlson (median (P_25_–P_75_))	2 (1–4)	3 (2–5)	<0.001
BMI (median (P_25_–P_75_))	31.2 (28.7–35.2)	30.9 (28.7–34.7)	0.545
Weight at discharge (median (P_25_–P_75_))	84 (75.5–99)	84 (75.2–99)	0.638
SBP after discharge (median (P_25_–P_75_))	130 (120–138)	130 (120–137)	0.223
DBP after discharge (median (P_25_–P_75_))	75 (70–80)	70 (65–80)	0.012

Note: Percentages were calculated with reference to the total value available for each variable.

**Table 2:** Distribution of diagnoses at admission (ICD-10).

Diagnosis Grouping (ICD 10)		Group		Total
		Intervention	Control	
G1 Certain infectious and parasitic diseases (A00–B99)	Count	6	1	7
	within group %	4.3%	0.8%	2.6%
G2 Neoplasms (C00–D49)	Count	2	6	8
	within group %	1.4%	4.5%	2.9%
G3 Diseases of the blood and blood-forming organs and certain disorders involving the immune mechanism (D50–D89)	Count	4	0	4
	within group %	2.8%	0.0%	1.5%
G4 Endocrine, nutritional, and metabolic diseases (E00–E89)	Count	4	1	5
	within group %	2.8%	0.8%	1.8%
G6 Other diseases of organs and body systems (G00–N99)	Count	102	106	208
	within group %	72.3%	80.3%	76.2%
G9 Various groups (R00–Z99)	Count	23	18	41
	within group %	16.3%	13.6%	15.0%
Total	Count	141	132	273
	within group %	100%	100%	100%

ICD 10: 10th revision of the International Classification of Diseases. G6 includes diseases with codes G00–G99, H00–H59, I00–I99, J00–J99, K00–K95, L00–L99, and M00–M99.

**Table 3:** Comparison of outcome variables between baseline and three months.

Variable	*n*	Baseline Mean ± SD	3 Months Mean ± SD	*p*-Value Time Effect	*p*-Value Group Effect	*p*-Value Time*Group Effect
Weight after discharge						
Intervention	88	87.62 ± 19.23	84.09 ± 16.59	<0.001	0.617	0.170
Control	69	85.31 ± 18.65	83.54 ± 18.28			
SBP after discharge						
Intervention	62	133.72 ± 19.60	109.37 ± 37.71	<0.001	0.187	<0.001
Control	57	127.42 ± 15.93	124.68 ± 13.94			
DBP after discharge						
Intervention	62	73.43 ± 15.06	63.90 ± 22.05	0.009	0.066	0.042
Control	57	73.17 ± 12.07	71.93 ± 11.25			
EQ-5D-5L value						
Intervention	90	0.71 ± 0.27	0.68 ± 0.30	0.035	0.589	0.563
Control	70	0.71 ± 0.24	0.65 ± 0.26			
VAS score (EQ-5D-5L)						
Intervention	84	58.19 ± 18.47	62.74 ± 22.63	0.046	0.310	0.983
Control	69	55.65 ± 20.93	60.10 ± 21.18			
Pardo Questionnaire						
CC dimension						
Intervention	83	2.21 ± 0.69	2.95 ± 0.68	<0.001	0.174	0.212
Control	63	2.17 ± 0.60	2.73 ± 0.88			
PW dimension						
Intervention	87	3.72 ± 1.07	4.03 ± 0.97	0.399	0.376	0.030
Control	68	4.06 ± 1.04	3.93 ± 1.01			
PE dimension						
Intervention	87	1.77 ± 0.92	1.78 ± 0.89	0.121	0.177	0.161
Control	68	1.79 ± 0.96	2.09 ± 1.20			
HD dimension						
Intervention	82	3.48 ± 0.57	4.40 ± 1.10	<0.001	0.881	0.017
Control	64	3.67 ± 0.59	4.19 ± 0.51			
AC dimension						
Intervention	88	3.45 ± 0.73	3.51 ± 0.79	0.133	0.406	0.021
Control	70	3.56 ± 0.76	3.26 ± 0.46			

Time*Group Effect = Between-group difference taking into account the time between baseline and three.month.

**Table 4:** Comparison of outcome variables between baseline and six months.

Variable	*n*	Baseline Mean ± SD	6 Months Mean ± SD	*p*-Value Time Effect	*p*-Value Group Effect	*p*-Value Time*Group Effect
Weight after discharge						
Intervention	85	89.93 ± 20.99	85.73 ± 19.09	<0.001	0.095	0.361
Control	65	84.45 ± 14.76	81.74 ± 13.91			
SBP after discharge						
Intervention	58	128.55 ± 15.01	121.41 ± 17.17	0.012	0.886	0.294
Control	53	126.09 ± 14.14	123.11 ± 21.95			
DBP after discharge						
Intervention	57	75.18 ± 10.38	67.79 ± 19.25	0.012	0.607	0.164
Control	53	71.58 ± 9.85	69.42 ± 14.15			
EQ-5D-5L value						
Intervention	86	0.71 ± 0.28	0.62 ± 0.34	0.001	0.482	0.970
Control	68	0.74 ± 0.25	0.65 ± 0.28			
VAS score (EQ-5D-5L)						
Intervention	82	56.8 ± 20.01	63.32 ± 23.09	0.018	0.451	0.587
Control	68	55.96 ± 20.58	60.06 ± 21.55			
Pardo Questionnaire						
CC dimension						
Intervention	81	2.22 ± 0.74	2.84 ± 0.71	<0.001	0.931	0.827
Control	65	2.21 ± 0.62	2.86 ± 0.99			
PW dimension						
Intervention	83	3.81 ± 1.05	3.84 ± 1.03	0.374	0.068	0.545
Control	63	4 ± 1.08	4.15 ± 0.87			
PE dimension						
Intervention	82	1.72 ± 0.94	2.07 ± 1.1	0.006	0.622	0.601
Control	66	1.85 ± 1.05	2.09 ± 1.32			
HD dimension						
Intervention	76	3.48 ± 0.62	4.2 ± 0.55	<0.001	0.101	0.755
Control	61	3.65 ± 0.62	4.33 ± 1.02			
AC dimension						
Intervention	83	3.42 ± 0.65	3.4 ± 0.63	0.065	0.970	0.106
Control	68	3.55 ± 0.79	3.28 ± 0.54			

Time*Group Effect = Between-group difference taking into account the time between baseline and six-month.

**Table 5:** Comparison of outcome variables between baseline and 12 months.

Variable	*n*	Baseline Mean ± SD	12 Months Mean ± SD	*p*-Value Time Effect	*p*-Value Group Effect	*p*-Value Time*Group Effect
Weight after discharge						
Intervention	73	87.87 ± 20.51	84.72 ± 17.58	<0.001	0.314	0.742
Control	66	84.98 ± 20.31	81.25 ± 18.45			
SBP after discharge						
Intervention	47	127.43 ± 12.91	121.28 ± 15.23	0.003	0.607	0.552
Control	43	127.56 ± 11.77	123.42 ± 12.21			
DBP after discharge						
Intervention	46	74.96 ± 8.64	70.76 ± 10.79	0.058	0.794	0.141
Control	43	72.69 ± 8.64	72.16 ± 10.28			
EQ-5D-5L value						
Intervention	74	0.74 ± 0.28	0.61 ± 0.35	<0.001	0.975	0.315
Control	66	0.71 ± 0.26	0.64 ± 0.33			
VAS score (EQ-5D-5L)						
Intervention	73	57.51 ± 19.25	59.30 ± 24.35	0.757	0.911	0.688
Control	64	58.2 ± 18.86	57.97 ± 25.54			
Pardo Questionnaire						
CC dimension						
Intervention	66	2.3 ± 0.73	3.20 ± 0.95	<0.001	0.415	0.699
Control	57	2.23 ± 0.63	3.07 ± 0.95			
PW dimension						
Intervention	72	3.94 ± 0.97	4.09 ± 0.87	0.151	0.05	0.941
Control	61	4.17 ± 0.92	4.32 ± 0.82			
PE dimension						
Intervention	71	1.8 ± 0.97	1.87 ± 1.32	0.735	0.703	0.830
Control	63	1.89 ± 1.07	1.91 ± 1.45			
HD dimension						
Intervention	68	3.41 ± 0.63	4.15 ± 0.66	<0.001	0.03	0.626
Control	59	3.68 ± 0.618	4.35 ± 0.41			
AC dimension						
Intervention	72	3.37 ± 0.712	3.29 ± 0.41	0.501	0.432	0.213
Control	64	3.61 ± 0.79	3.27 ± 0.48			

Time*Group Effect = Between-group difference taking into account the time between baseline and twelve-month.

**Table 6:** Comparison of readmissions, emergency department visits, and deaths during the follow-up.

Variable	0–3 Months		3–6 Months		6–12 Months	
**Readmissions**						
	*n*	Readmissions	*n*	Readmissions	*n*	Readmissions
Intervention	125	32 (25.6%)	113	12 (10.6)	104	18 (17.3%)
Control	111	25 (22.5%)	96	10 (10.4%)	92	20 (21.7%)
*p* 0–3 m = 0.690; *p* 3–6 m = 1; *p* 6–12 m = 0.547						
**Visits to the emergency department**						
	*n*	Visits to the emergency department	*n*	Visits to the emergency department	*n*	Visits to the emergency department
Intervention	125	47 (37.6%)	113	29 (25.7%)	104	26 (25%)
Control	111	38 (34.2%)	96	27 (27.6%)	92	26 (28.3%)
*p* 0–3 m = 0.688; *p* 3–6 m = 0.878; *p* 6–12 m = 0.723						
**Death**						
	*n*	Deaths	*n*	Deaths	*n*	Deaths
Intervention	125	9 (7.4%)	113	9 (8%)	104	9 (8.7%)
Control	111	16 (14.5%)	96	4 (4.2%)	92	5 (5.4%)
*p* 0–3 m = 0.079; *p* 0–6 m = 0.257; *p* 6–12 m = 0.382						

**Table 7:** CCI ≥ 3. Comparison between baseline and 12 months (*n* = 151).

Variable	*n*	Baseline Mean ± SD	12 Months Mean± SD	*p*-Value Time Effect	*p*-Value Group Effect	*p*-Value Time*Group Effect
Weight after discharge						
Intervention	27	83.75 ± 16.80	83.63 ± 17.04	0.069	0.220	0.0893
Control	48	80.43 ± 18.83	76.95 ± 15.95			
SBP after discharge						
Intervention	19	130.63 ± 15.55	121.05 ± 15.60	0.001	0.826	0.407
Control	34	128.03 ± 12.98	122.26 ± 11.67			
DBP after discharge						
Intervention	19	75.74 ± 7.30	70.53 ± 12.35	0.046	0.427	0.121
Control	34	71.65 ± 8.69	70.97 ± 9.22			
EQ-5D-5L value						
Intervention	27	0.61 ± 0.31	0.43 ± 0.34	0.001	0.098	0.267
Control	48	0.67 ± 0.25	0.58 ± 0.32			
VAS score (EQ-5D-5L)						
Intervention	26	52.43 ± 17.28	56.04 ± 24.26	0.514	0.341	0.667
Control	47	57.55 ± 18.62	58.30 ± 22.58			
Pardo Questionnaire						
CC dimension						
Intervention	25	2.42 ± 0.57	3.53 ± 0.92	<0.001	0.086	0.163
Control	44	2.28 ± 0.67	3.07 ± 0.98			
PW dimension						
Intervention	26	4.10 ± 1.05	4.29 ± 0.81	0.431	0.447	0.615
Control	47	4.29 ± 0.83	4.33 ± 0.79			
PE dimension						
Intervention	25	1.73 ± 0.90	1.57 ± 1.22	0.644	0.307	0.968
Control	46	1.99 ± 1.12	1.82 ± 1.42			
HD dimension						
Intervention	25	3.81 ± 0.47	4.26 ± 0.58	<0.001	0.711	0.313
Control	43	3.75 ± 0.59	4.39 ± 0.40			
AC dimension						
Intervention	26	3.40 ± 0.87	3.17 ± 0.37	0.005	0.352	0.519
Control	47	3.59 ± 0.79	3.22 ± 0.45			

Time*Group Effect = Between-group difference taking into account the time between baseline and twelve-month.

## Data Availability

The underlying data are available on request.

## Supplementary Materials

The following are available online at https://www.mdpi.com/article/10.3390/nu14122499/s1, Table S1: CONSORT 2010 checklist of information to include when reporting a randomised trial*, Supplementary File S1: General Recommendations (control group); Supplementary File S2: Intervention.
