# Supplementary material for: Hospital Intervention to Reduce Overweight with Educational Reinforcement after Discharge: A Multicenter Randomized Clinical Trial
Source: Nutrients. 2022 Jun 16;14(12):2499. doi: 10.3390/nu14122499 (PMC9227976; doi:10.3390/nu14122499)
Supplement: Supplementary file 1 [file nutrients-14-02499-s001.zip › nutrients-1733912-supplementary/Supplementary File 1 General Recommendations (control group).docx]

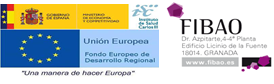


**Overweight treatment: General Recommendations**

Reduce the consumption of sugars and fats

Reduce food portions

Increase fiber intake

Drink more water

Reduce alcohol and carbonated drinks

Increase physical activity

Research Project

EFFECTIVENESS AND COST-USEFULNESS OF A HOSPITAL INTERVENTION TO REDUCE OBESITY, WITH EDUCATIONAL REINFORCEMENT AFTER DISCHARGE

Carmen Herrera Espiñeira et al
