# Supplementary material for: Hospital Intervention to Reduce Overweight with Educational Reinforcement after Discharge: A Multicenter Randomized Clinical Trial
Source: Nutrients. 2022 Jun 16;14(12):2499. doi: 10.3390/nu14122499 (PMC9227976; doi:10.3390/nu14122499)

Advice on diet and exercise for weight loss

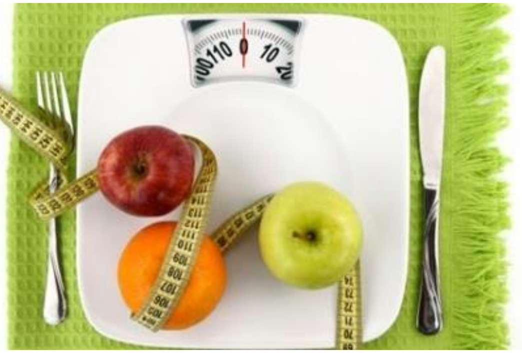

Research project

EFFECTIVENESS AND COST-USEFULNESS OF A HOSPITAL INTERVENTION TO REDUCE OBESITY, WITH EDUCATIONAL REINFORCEMENT AFTER DISCHARGE

Carmen Herrera Espiñeira et al

You have just been through a severe process. It is in your hands to modify your weight so that these episodes are milder or do not appear.

Your health is influenced by your weight, which is affected by your diet and physical activity. Excess eating can be influenced by situations that worry you and by a sedentary life with very few activities that interest you

4 moments are crucial: THE 4 Cs.
COMPRAR (BUYING) the food
COCINAR (COOKING)
COMER (EATING)
CAMINAR (WALKING)

BUYING

- Look at the correct proportion of foods in the picture
- Buy when you are not hungry
- Reduce the purchase of sweets and cold meats and
- Keep them at home in places that are not visible
- Buy semi-skimmed milk and derivatives
- Buy more vegetables and pulses
- Do not buy prepared food (has a lot of salt and sugar)
- Do not buy carbonated or sugared drinks

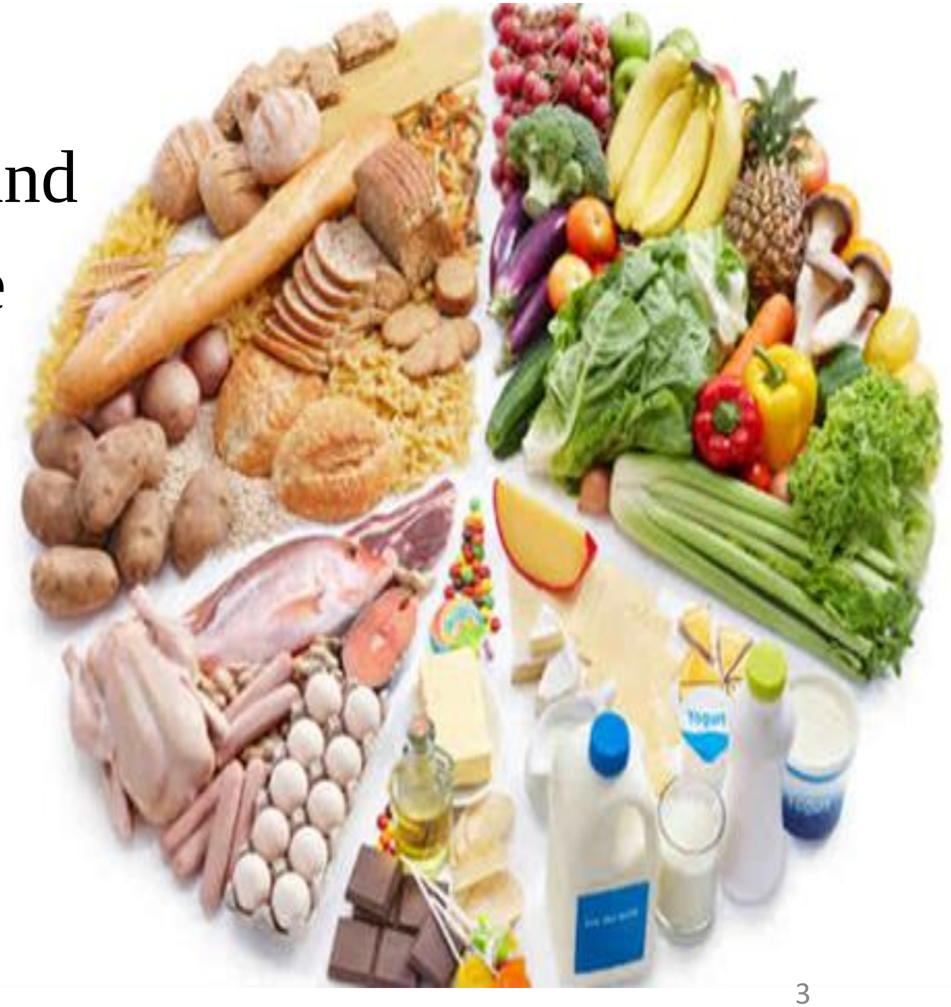

COOKING

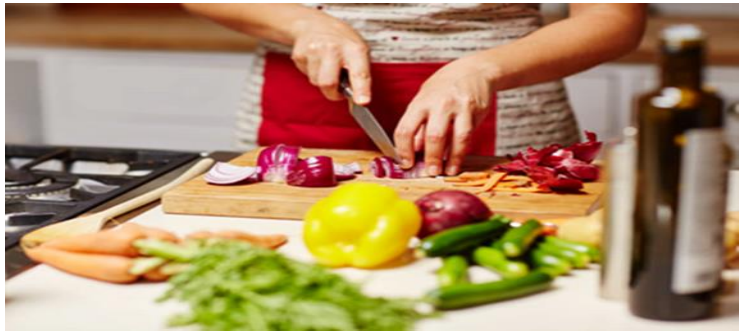

- Do not cook when you are hungry
- Reduce the salt in foods. Replace it with spices (curry, curcumin, oregano, thyme, lemon, …)
- Cook with the grill, steamer, oven, preferably with little sauce/gravy
- Use less oil. De-fat broths and soups, removing visible fat.
- Prepare hot products (give a greater feeling of satiation)
- Prepare vegetables for all meals: fresh or boiled
- Cook mainly fish, chicken, or turkey
- Prepare desserts in which sugar is replaced with artificial sweeteners or use sweet spices such as cinnamon, vanilla…

EATING

- Have 3 main meals and 2 snacks
- (this avoids coming to main meals feeling very hungry)

- Eat sitting down
- Do not eat or drink before meals
- Do not mix dishes
- Eat slowly (foods will swell more and you will feel satiated earlier)

EATING

1 BREAKFAST: toast, skimmed dairy products, and fruit

2 Mid-morning snack: fruit, skimmed yogurt, or vegetable such as carrot

3 LUNCH: Eat the vegetables first (the food will expand more in your stomach and you will feel less hungry)

-Half-fill the plate with main dish or use a small plate and large glass of water

-Reduce the amount of alcoholic drinks

-Do not drink carbonated drinks (they dilate your stomach and contain salt)

-Serve the food and remove the platter from the table

-Leave the table once you finish eating

4 Mid-afternoon snack: coffee with milk, yogurt, fruit

5 DINNER/SUPPER: The same indications as for lunch

Have a small dinner and not immediately before sleeping (while you sleep your organism will preferentially consume fats)

Avoid eating sandwiches

WALKING and other physical exercises

- Preferably walk with someone else (you exercise more without noticing). Perform arm movement exercises, more often if you cannot walk much

- If you add resistance exercises besides walking (swimming, cycling, climbing stairs..) you will improve your musculature and bone density (especially important in women)

- Gradually increase the duration of walking and other exercises

- BENEFITS
- Increases energy expenditure and controls the appetite
- Reduces obesity-related disease risk factors
- Assists in combatting anxiety and stress
- You carry out activities that are fun

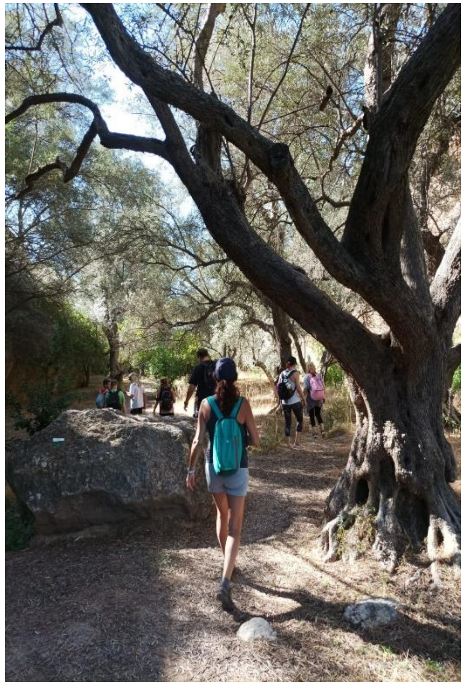

Supplement: Supplementary file 1 [file nutrients-14-02499-s001.zip › nutrients-1733912-supplementary/Supplementary File 2 Intervention.pdf]
